# Virus‐Surface‐Mimicking Surface Clustering of AuNPs onto DNA‐Entrapped Polymeric Nanoparticle for Enhanced Cellular Internalization and Nanocluster‐Induced NIR Photothermal Therapy

**DOI:** 10.1002/advs.201500108

**Published:** 2015-07-14

**Authors:** Hui‐Zhen Jia, Wei‐Hai Chen, Xuli Wang, Qi Lei, Wei‐Na Yin, Yan Wang, Ren‐Xi Zhuo, Jun Feng, Xian‐Zheng Zhang

**Affiliations:** ^1^ Key Laboratory of Biomedical Polymers of Ministry of Education and Department of ChemistryWuhan UniversityWuhan430072P.R. China; ^2^ Department of Phamaceutics and Pharmaceutical ChemistryUniversity of UtahSalt Lake CityUT84108USA; ^3^ Institute of HydrobiologyChinese Academy of Sciences Analysis and Testing Center430072WuhanP.R. China

**Keywords:** cellular entry, gene transfection, gold nanoparticles, photothermal therapy, virus‐surface mimicking

## Abstract

**Virus‐surface‐mimicking decoration of deoxyribonucleic acid (DNA)‐entrapped polymeric nanoparticle with AuNPs** is demonstrated to lead to enhanced cellular uptake, improved gene transfection, and particularly efficient near‐infrared photothermal therapy that cannot be achieved by both of them separately. This hybrid nanosystem represents a novel paradigm of multipurpose organic–inorganic nanoplatform, especially for cancer treatments.

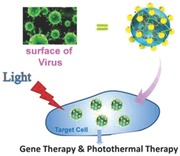

Gene therapy has shown great potency for treating various diseases, such as cancers.^1^ One urgent task for gene therapy is focused on how to enable efficient transport of therapeutic genes into host cells, for which the assistance of suitable vectors is required.^2^, ^3^, ^4^ Nowadays, polycationic vectors have been among the most promising nonviral candidates for gene delivery,^5^ but their transfection activities seem far from satisfactory and need to be improved. Because the cell entry of DNA/vector nanocomplex is one of the crucial factors affecting the overall transfection performance, universal strategies that can promote the cellular uptake are highly desired.^6^ Up‐to‐date advance in understanding the structure–function relationship of enveloped viruses offers useful information to guide the design of artificial vectors, which opens possibilities of improving the transmembrane ability to a high level.^7^ Many viruses such as influenza virus, herpes simplex virus (HSV),^8^ and human immunodeficiency virus (HIV)^9^ are identified to possess a rough surface patched by glycoprotein spikes, as shown in **Figure** **1**. The nanoscale roughness of viral surface caused by glycoprotein spikes is thought to be “friendly” to cellular membrane and benefit the cell entry.^10^, ^11^ It is further found that the sparsely distributed glycoproteins spikes would voluntarily cluster together to facilitate the viral entry into cells.^12^

**Figure 1:**
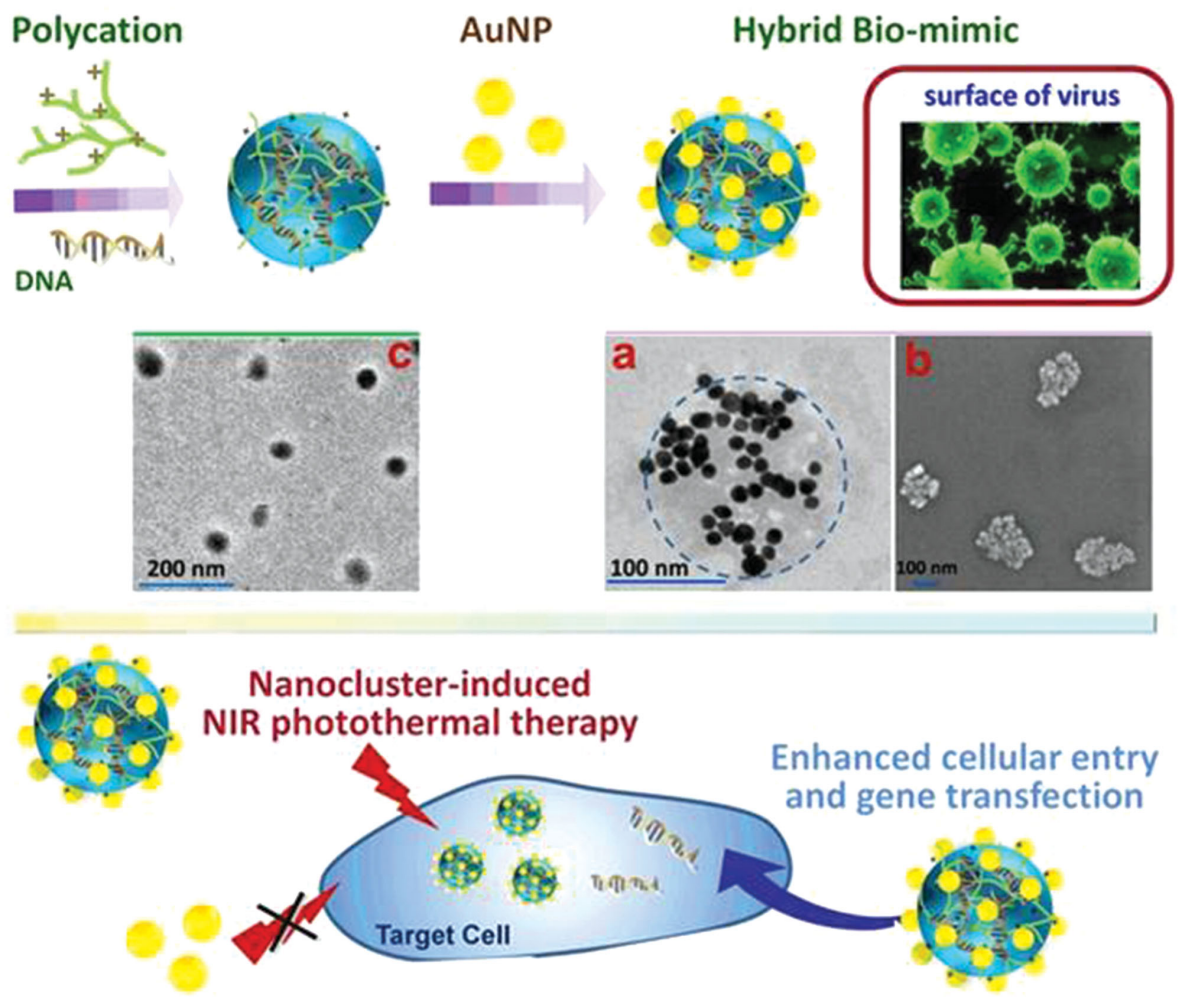
Illustration of the virus‐surface‐mimicking hybrid decoration of DNA‐entrapped nanoparticle with AuNPs for enhanced gene transfection and nanocluster‐induced NIR photothermal therapy and TEM image of PEI1800/pGL‐3/AuNP nanoparticles (inset (a)) as well as SEM image of PEI1800/pGL‐3/AuNP nanoparticles (inset (b)) and TEM images of PEI1800/pGL‐3 (inset (c)).

Drawing lessons from the viral topography, the present work reported a virus‐surface‐mimicking nanotechnology by decorating gene‐entrapped polymeric nanoparticles with clustered gold nanoparticles (AuNPs) (Figure 1). This bio‐mimic design has demonstrated significantly enhanced cellular internalization of DNA payloads and consequently up to 100‐fold promotion of transfection efficacy. Inorganic nanoparticles, particularly AuNPs, have become a hot research area in the last decade; however, most of the reported studies are focused on exploiting them as the nanomatrix to undergo surface modification with hydrophilic polymers or biomolecules for biomedical applications such as in vivo imaging and photothermal therapy.^13^, ^14^ Differently, the strategy of using AuNPs to modify organic nanosystem has nearly not been described. More attractively, this hybridization strategy paves a special avenue to realize the from‐no‐to‐yes hyperthermia induction of AuNPs in the near‐infrared (NIR) region. As known, one significant challenge for AuNP‐induced photothermal therapy is that AuNPs, particularly spherical ones, mainly absorb light in the visible range with a more shallow penetration depth in tissue as compared to the therapeutic window in the NIR region. Several studies attract our interest that the AuNP aggregates self‐assembled on cell surface can effectively increase the photothermal efficacy under NIR irradiation.^15^, ^16^, ^17^ It is supposed that the 3D location of AuNP clusters on the surface of DNA/vector nanocomplex can produce this exciting feature, as actually proved herein with high efficiency to kill cancerous cells, in contrast to the failure of both of them separately. This hybrid nanosystem represents a novel paradigm of gene‐based multipurpose nanoplatform and can be extended to the convenient engineering of many hybrid inorganic–organic nanoplatforms with versatile multifunctions.

In a proof‐of‐principle experiment, a positively charged nanocomplex of plasmid pGL‐3 condensed with lowly toxic polyethylenimine (PEI1800, *M*_w_ = 1800 Da) was prepared at the optimal transfection N:P ratio of 20:1 and was used as the organic nanomatrix for the sequent fabrication of hybrid NPs. The nanocomplex possessed the surface zeta potential of +27 mV and the mean hydrodynamic diameter of 110 nm, as determined by dynamic light scattering (DLS; Figure S1, Supporting Information). Citrate‐coated AuNP (particle size »10 nm, zeta potential approximately –35.8 mV) was slowly added into the nanocomplex solution under vibration. Driven by electrostatic interaction, the nanocomplex would readily be covered by a high density of smaller AuNPs (Figure 1). To simplify the research, the mass ratio of AuNP versus nanocomplex was optimized and fixed at 7:1 throughout the study, based on the in vitro transfection experiment in HeLa cells (as discussed later). Transmission electron microscopy (TEM) and scanning electron microscopy (SEM) strongly evidenced that AuNPs can bind to the nanocomplex and cluster into a rough periphery. TEM image showed that parent PEI1800/DNA nanocomplex displayed a regularly spherical shape with smooth surface and the particle size was around 50 ± 3 nm in dry state (Figure 1, inset (c)). In comparison, the addition of AuNPs led to a marked change of the size and topography of the nanoparticles. As seen from the typical SEM image, the formed PEI1800/pGL‐3/AuNP nanoparticles still remained individually dispersed whereas possessing an apparently rough surface (Figure 1, inset (b)). The magnified TEM image obtained at a lower dosage of AuNPs offered information to better understand the morphological changes, showing that the roughened surface was ascribed to the attachment of lots of AuNPs on nanocomplex (Figure 1, inset (a), small black dots on nanoparticles surface). As a result, the particle size was increased to about 200 ± 12 nm, which was slightly smaller than the hydrodynamic diameter of 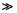
225 ± 11 nm. Meanwhile, AuNP attachment induced a sharp decline of zeta potential from 27.0 ± 1.0 mV to 5.0 ± 0.4 mV (Table S1, Supporting Information), reconfirming the successful manufacture of hybrid PEI1800/pGL‐3/AuNP nanoparticles. Upon AuNP addition, the enhanced hydrodynamic dia­meter of PEI1800/DNA naocomplex might associate with the charge neutralization occurring between the nanocomplex and the opposite charged AuNPs. As a consequence, the nanocomplex may be less compact than that without AuNP decoration.

To verify the feasibility of our virus‐surface‐mimicking idea, the intracellular internalization and the in vitro transfection of the AuNP‐absent and ‐present nanocomplexes were comparatively explored. Four types of cell lines including human cervical cancer (HeLa) cells, monkey kidney fibroblast (COS7) cells, human hepatoma (HepG2) cells, and mouse fibroblast (3T3) cells were tested to evaluate the universal adaptability of this strategy. Confocal laser scanning microcopy (CLSM) provided an intuitive inspection over the cellular uptake of YOYO‐1‐labeled plasmid pGL‐3. As shown in **Figure** **2**A, the green fluorescence with strong intensity appeared in all the cell lines after the cells were exposed to PEI1800/pGL‐3/AuNP for 4 h. In comparison, the treatments with AuNP‐absent nanocomplex gave much lower cell‐entry efficiencies of entrapped DNA, as reflected by the considerably weakened fluorescence inside cells. Such an entry promotion caused by AuNP decoration appeared to be general, regardless of the investigated cell types. Moreover, quantitative analysis using fluorescence‐activated cell sorting offered solid data regarding the ability of delivering pGL‐3 into cells. In consistence with the CLSM observation, the obtained profiles showed that the amount of YOYO‐1‐positive cells when exposed to PEI1800/pGL‐3‐YOYO‐1/AuNP was at a much higher level than that treated with the counterpart of PEI1800/pGL‐3‐YOYO‐1 (Figure 2B). Further comparison in the mean fluorescence intensity indicated that the cellular delivery efficiency of the former was threefold to sevenfold than that of the latter (Figure 2B). Among four cell lines, COS7 cells were shown to be less sensitive to PEI1800/pGL‐3‐YOYO‐1 nanocomplex, leading to much lower uptake efficiency. Interestingly, the AuNP‐induced promotion effect was represented more profoundly in COS7 cells with the highest sevenfold promotion.

**Figure 2:**
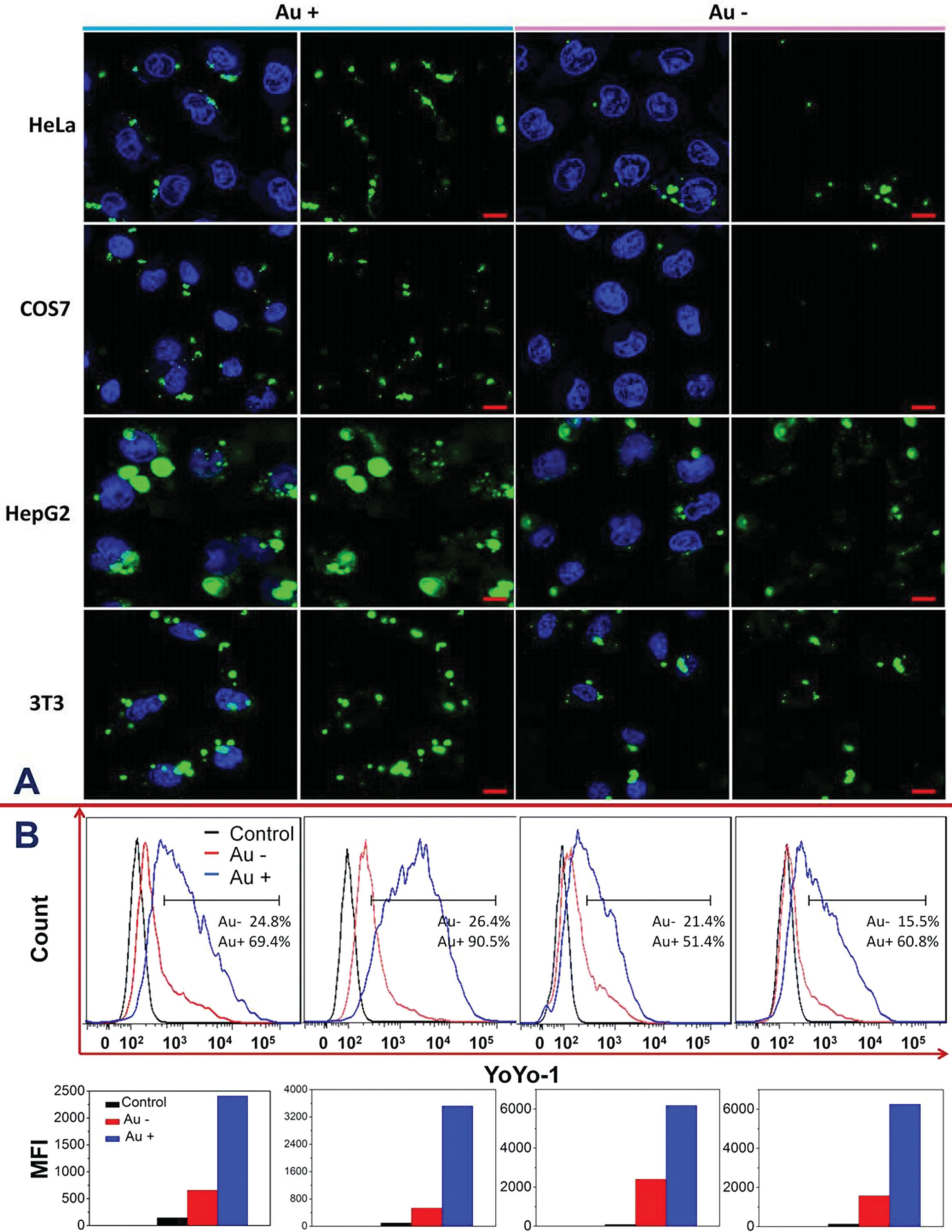
A) Confocal images of various cell lines after the treatment with AuNP‐absent (Au−) or AuNP‐present (Au+) PEI1800/pGL‐3 nanocomplexes. The concentration of plasmid pGL‐3 was fixed at 1 μg mL^−1^. pGL‐3 were stained green by YOYO‐1 and nuclei were stained blue by Hoechst 33342. The scale bar was 20 μm. B) Flow cytometry profiles obtained after the treatment with PEI1800/pGL‐3 and PEI1800/DNA/AuNP (upper lane) as well as the comparison of the mean fluorescence intensity inside cells (lower lane). Blank cells were selected as the control. The mass ratio of AuNP versus DNA/vector nanocomplex was fixed at 7:1.

The electrostatic attractiveness of cell membrane to polycation/DNA nanocomplex plays a predominant role for its cellular entry. In principle, higher charge of positive nanocomplex benefits the intracellular delivery though it usually gives rise to the damages to cells.^18^ As described above, AuNP attachment onto the nanocomplex reduced the zeta potential from 27 mV down to a neutral‐close level of 5 mV. When taking into account the sharp decline of zeta potential, the observed enhancement of delivery efficiency resulting from AuNP attachment seems confusing. There ought to exist other factors that affect the intracellular transport of carried DNA and thereby complement the sacrifice caused by the reduced surface charge. Recent studies have identified that the rough surface patched by glycoprotein spikes constitutes a “nanoecology” topography for many enveloped viruses, which is thought to contribute positively to their cell entry.^19^ This speculation can find the evidence that the silica nanoparticle displayed a substantial promotion of cellular entry efficiency after calcinating relatively smaller ones onto its surface.^11^ Likewise, the easier cellular internalization of the AuNP‐attached nanocomplex might associate with the resulting roughness of nanocomplex topography though the definite mechanism may be more complicated.

Being aware that the surficial AuNP clustering can facilitate the cell uptake of PEI800/pGL‐3 nanocomplex, it is interesting to make clear the resulting influence on the transfection performance. The in vitro transfection was therefore studied by luciferase assay in the 10% serum‐containing medium. As expected, AuNP attachment made a positive impact on the transfection so that the protein expression level was dramatically enhanced in all of the four transfected cell lines at the mass ratio of AuNP versus nanocomplex of 7:1 (Figure S2B, Supporting Information). As far as the promotion effect of pGL‐3 protein expression was concerned, the best outcome was detected in COS7 cells with approximately a 100‐fold enhancement. This correlated well with the finding that the AuNP‐induced enhancement of cell uptake was more distinguished in COS7 cells, validating the feasibility of improving the transfection by virtue of promoting the intracellular plasmid transport. Enhanced green fluorescent protein plasmid (pEGFP‐C1) was also used as the reporter gene for the transfection tests and the transfected cells were observed by CLSM. Compared with the controls using PEI1800/pEGFP‐C1 nanocomplex, inside the cells transfected with PEI1800/pEGFP‐C1/AuNP appeared significantly stronger green fluorescence regardless of the cell types (**Figure** **3**A), which agreed well with the quantitative lucerifase assay using pGL‐3 reporter gene.

**Figure 3:**
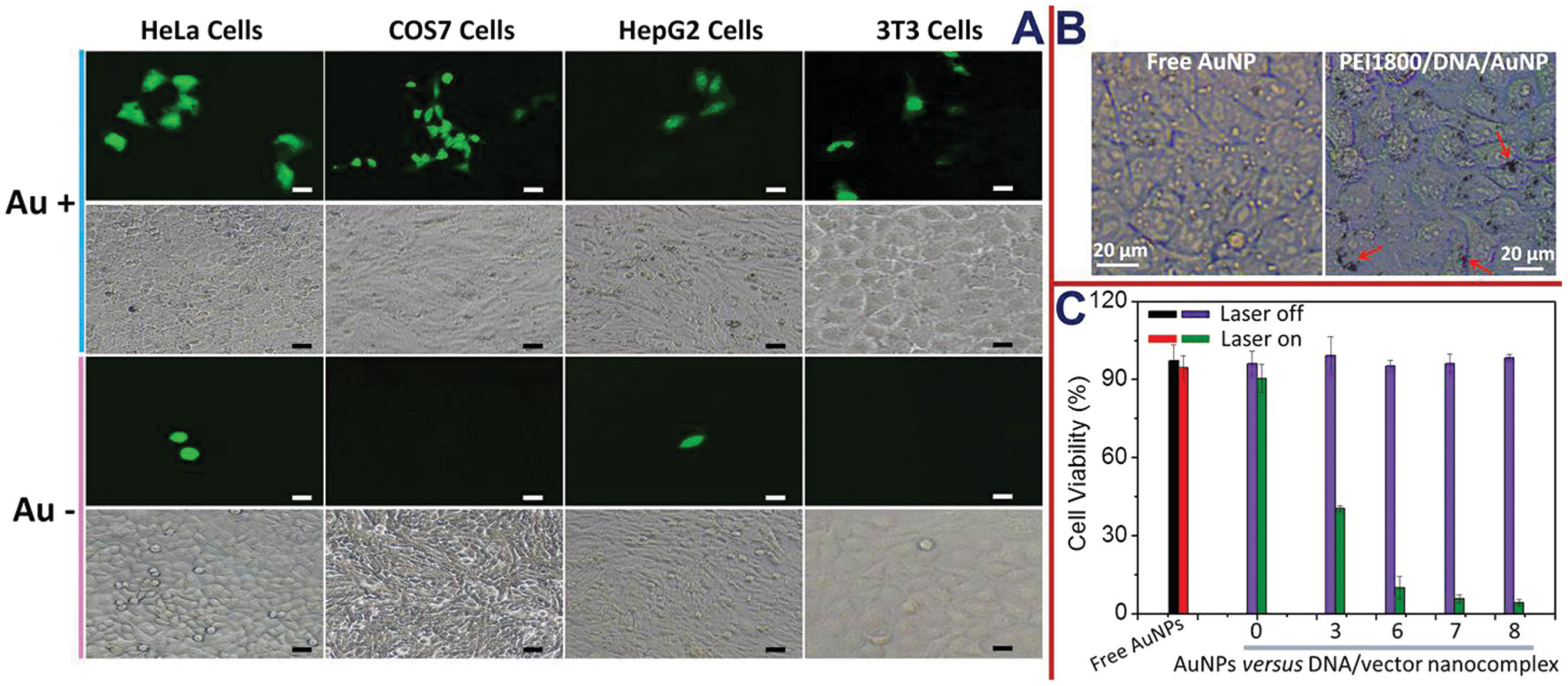
A) Microphotographs of enhanced green fluorescent protein expression mediated by PEI1800/pEGFP‐C1 and PEI1800/pEGFP‐C1/AuNP. The concentration of pEGFP‐C1 was fixed at 1 μg mL^−1^. The w/w ratio of AuNP versus DNA/vector nanocomplex was fixed at 7:1. B) CLSM images of the HeLa cells after the incubation with free AuNPs or PEI1800/DNA/Au nanoparticles. The weight ratio of AuNP versus nanocomplex was 7:1.C) Photothermal effects in HeLa cells. 1 min irradiation was applied to cells after the coincubation with samples at various w/w ratios of AuNP versus DNA/vector nanocomplex for 20 h. The laser power was 2 W cm^−2^ and the concentration of HeLa cells was 5.0 × 10^4^ cells per well; the N/P ratio of PEI1800 versus pGL‐3 was 20, the pGL‐3 concentration was fixed at 1 μg mL^−1^.

The relationship between the transfection efficacy and the introduced AuNP dosage has been explored (Figure S2A, Supporting Information). It was shown that as elevating the weight ratio (AuNP vs nanocomplex), the transfection efficacy took on a gradual increment followed by a plateau starting from the ratio at around 6:1. This finding was consistent with the obtained DLS data in terms of the zeta potential. It was found that the variation profile of the zeta potential also involved two stages, where the zeta potential intended to be insignificantly changed after the ratio reached the same ratio (Figure S3B, Supporting Information). Theoretically, the topography of PEI800/pGL‐3/AuNP nanoparticles would be slightly affected when the surficial AuNP attachment is close to saturation. Thereafter, further increasing AuNP amount would make few influences on the surface potential and the transfection efficiency. Based on the obtained results, therefore, a relatively higher ratio of 7:1 was exploited throughout this study to ensure the complete coverage of the complex surface with AuNPs. On the other hand, DLS data showed that the diameter of the hybrid NPs kept slowly growing along with the ratio increase. The continuous increase of the particle diameter from 210 to 270 nm after the ratio reached 6:1 may possibly associate with the minor aggregation at a higher AuNP dosage (Figure S3A, Supporting Information). However, such a slight change in the diameter appeared to insignificantly affect the transfection efficacy (Figure S2, Supporting Information).

A tetrazolium dye (MTT) based assay was carried out to study the influence of AuNP attachment on the cell toxicity of PEI1800/pGL‐3 nanocomplex. As shown in Figure S4 (Supporting Information), the cell proliferation was nearly not affected by AuNP attachment. The relative cell viability of AuNP‐free and ‐present nanocomplexes always remained above 90% in all of the four cell lines, suggesting the low cytotoxicity originating from the good biocompatibility of PEI1800 and AuNPs.

Rapidly proliferating tumor cells are more sensitive to heat shock than slowly proliferating cells through the mechanisms such as mitotic delay, cell cycle arrest, and plasma membrane damage.^20^ Several studies have shown that the performance of NIR photothermal therapy is sensitive to the size and aggregation state of the gold nanoparticles. Au clusters can elicit coupled surface plasmons and strong absorption at NIR wavelengths with efficient heat conversion, in contrast to the plasmon resonance of free spherical AuNPs within visible range.^21^, ^22^ It has been identified that gold nanoshells, which was arranged to cover inorganic silica/Fe3O4 nanocore, can offer strengthened NIR absorption, efficiently convert the light into localized heat, and thus effectively kill cancer cells.^23^, ^24^ Also, AuNP aggregates self‐assembled on cell surface were found to offer an increased photothermal efficacy under NIR irradiation.^25^ In our hybrid nanosystem, “gold nanoclusters” can be formed, where many AuNPs are clustered together on the nanocomplex surface, creating opportunities for photothermal therapy with enhanced NIR absorption efficiency. To verify this, cancerous HeLa cells were used as the typical model and the cell viability was monitored upon the exposure to CW diode laser irradiation at 808 nm,^26^ which is in the optimal penetration window of most biological tissues.^27^ All the experiments were conducted under the identical conditions and the cells were coincubated with the samples for 20 h prior to the irradiation taken with a very short period of 1 min. It was found that the irradiation treatment imparted negligible influences on the cell proliferation when the cells were exposed to either free AuNP or parent PEI1800/pGL‐3 nanocomplex, indicating the incapability of them for photothermal induction under the condition (Figure 3C). Oppositely, the exposure to irradiation led to a robust cell‐killing effect when the cells were treated with PEI1800/pGL‐3/AuNP, revealing that the success of NIR phototherapy totally relied on the achievement of clustering AuNP on PEI1800/pGL‐3 nanocomplex. The weight ratios above 6:1 (AuNP vs DNA/vector nanocomplex) were sufficient to kill almost all of the HeLa cells. The result correlated fairly well with the aforementioned relevance of the transfection efficiency and zeta potential with the weight ratio, corroborating the surface‐saturation hypothesis from another perspective. Furthermore, it was suggested that the majority of the AuNPs in the presence of PEI1800/pGL‐3 nanocomplex were actually in a sharply different state from free AuNPs, as can be further demonstrated by CLSM images (Figure 3B). As shown, inside the cells exposed to PEI1800/pGL‐3/AuNP (*W*_AuNP_:*W*_complex_ = 7:1) appeared lots of dark spots representing the clustered AuNPs that cannot be detectable at all for the treatment using free AuNP. In addition, the comparison between TEM images revealed that a portion of AuNPs in the PEI1800/pGL‐3/AuNP system still remained in the free state. To a certain extent, this finding implied that the weight ratio of 7:1 used in this study can guarantee the saturated coverage of the nanocomplex with AuNPs.

In summary, a virus‐surface‐mimicking nanotechnology was herein proposed to develop nonviral gene delivery nanosystem by electrostatically coating DNA‐entrapped polymeric nanoparticles with AuNPs. The resulting rough periphery composed of AuNP nanoclusters led to the significantly enhanced cellular uptake of DNA payload and consequently up to 100‐fold promotion of transfection efficacy as compared with the AuNP‐absent counterpart. Attractively, this hybridization approach allows the facile import of hyperthermia‐induction capability into the traditional gene delivery nanosystem, as demonstrated herein by the outstanding potency for the NIR photothermal therapy in cancerous HeLa cells. It is noted that this distinguished advantage highly relied on the success to form gold “nanoclusters” on the hybrid nanosystem, whereas both the parent nanocomplex and the AuNP separately failed under the identical NIR treatments. Further interests toward this nanotechnology would arise from its expandability to other inorganic NPs for the establishment of multipurpose gene‐based nanoplatforms, e.g., by using iron oxide NPs to combine gene therapy with the magnetic‐induced targeting and thermal therapy.

## Supporting information

As a service to our authors and readers, this journal provides supporting information supplied by the authors. Such materials are peer reviewed and may be re‐organized for online delivery, but are not copy‐edited or typeset. Technical support issues arising from supporting information (other than missing files) should be addressed to the authors.

Supplementary

## References

[1] K. Miyata , N. Nishiyama , K. Kataoka , Chem. Soc. Rev. 2012, 41, 2562. 22105545 10.1039/c1cs15258k

[2] C. Y. Lai , C. M. Wiethoff , V. A. Kickhoefer , L. H. Rome , G. R. Nemerow , ACS Nano 2009, 3, 691. 19226129 10.1021/nn8008504 PMC2707358

[3] L. Yan , J. F. Zhang , C. S. Lee , X. F. Chen , Small 2014, 10, 4487. 25168360 10.1002/smll.201401532

[4] C. E. Thomas , A. Ehrhardt , M. A. Kay , Nat. Rev. Genet. 2003, 4, 346. 12728277 10.1038/nrg1066

[5] H. Yin , R. L. Kanasty , A. A. Eltoukhy , A. J. Vegas , J. R. Dorkin , D. G. Anderson , Nat. Rev. Genet. 2014, 15, 541. 25022906 10.1038/nrg3763

[6] A. F. Adler , K. W. Leong , Nano Today 2010, 5, 553. 21383869 10.1016/j.nantod.2010.10.007 PMC3048656

[7] P. Zhu , J. Liu , J. Bess , E. Chertova , J. D. Lifson , H. Grisé , G. A. Ofek , K. A. Taylor , K. H. Roux , Nature 2006, 441, 847. 16728975 10.1038/nature04817

[8] K. Grünewald , P. Desai , D. C. Winkler , J. B. Heymann , D. M. Belnap , W. Baumeister , A. C. Steven , Science 2003, 302, 1396. 14631040 10.1126/science.1090284

[9] J. Chojnacki , T. Staudt , B. Glass , P. Bingen , J. Engelhardt , M. Anders , J. Schneider , B. Müller , S. W. Hell , H. G. Kräusslich , Science 2012, 338, 524. 23112332 10.1126/science.1226359

[10] C. LoPresti , M. Massignani , C. Fernyhough , A. Blanazs , A. J. Ryan , J. Madsen , N. J. Warren , S. P. Armes , A. L. Lewis , S. Chi‐rasatitsin , A. J. Engler , G. Battaglia , ACS Nano 2011, 5, 1775. 21344879 10.1021/nn102455z

[11] Y. Niu , M. H. Yu , S. B. Hartono , J. Yang , H. Y. Xu , H. W. Zhang , J. Zhang , J. Zou , A. Dexter , W. Y. Gu , C. Z. Yu , Adv. Mater. 2013, 25, 6233. 23946251 10.1002/adma.201302737

[12] G. Simmons , J. D. Reeves , A. J. Rennekamp , S. M. Amberg , A. J. Piefer , P. Bates , Proc. Natl. Acad. Sci. USA 2004, 101, 4240. 15010527 10.1073/pnas.0306446101 PMC384725

[13] D. A. Giljohann , D. S. Seferos , W. L. Daniel , M. D. Massich , P. C. Patel , C. A. Mirkin , Angew. Chem. Int. Ed. 2010, 49, 3280. 10.1002/anie.200904359 PMC3930332 20401880

[14] C. S. Kim , G. Y. Tonga , D. Solfiell , V. M. Rotello , Adv. Drug Delivery Rev. 2013, 65, 93. 10.1016/j.addr.2012.08.011 PMC3538170 22981754

[15] V. Zharov , R. Letfullin , E. Galitovskaya , J. Phys. D: Appl. Phys. 2005, 38, 2571.

[16] A. Y. Lin , J. K. Young , A. V. Nixon , R. A. Drezek , Small 2014, 10, 3246. 24729414 10.1002/smll.201303593

[17] S. Son , J. Nam , J. Kim , S. Kim , W. J. Kim , ACS Nano 2014, 8, 5574. 24869928 10.1021/nn5022567

[18] V. S. Trubetskoy , S. C. Wong , V. Subbotin , V. G. Budker , A. Loomis , J. E. Hagstrom , J. A. Wolff , Gene Ther. 2003, 10, 261. 12571634 10.1038/sj.gt.3301888

[19] A. Harris , G. Cardone , D. C. Winkler , J. B. Heymann , M. Brecher , J. M. White , A. C. Steven , Proc. Natl. Acad. Sci. USA 2006, 103, 19123. 17146053 10.1073/pnas.0607614103 PMC1748186

[20] G. Maldonado‐Codina , S. Llamazares , D. M. Glover , J. Cell Sci. 1993, 105, 711. 8408298 10.1242/jcs.105.3.711

[21] E. Boisselier , D. Astruc , Chem. Soc. Rev. 2009, 38, 1759. 19587967 10.1039/b806051g

[22] A. Kumar , S. Kumar , W. K. Rhim , G. H. Kim , J. M. Nam , J. Am. Chem. Soc. 2014, 136, 16317. 25386786 10.1021/ja5085699

[23] H. Y. Liu , D. Chen , L. L. Li , T. L. Liu , L. F. Tan , X. L. Wu , F. Q. Tang , Angew. Chem. Int. Ed. 2011, 123, 921.

[24] Z. Fan , M. Shelton , A. K. Singh , D. Senapati , S. A. Khan , P. C. Ray , ACS Nano 2012, 6, 1065. 22276857 10.1021/nn2045246 PMC3289758

[25] M. Everts , V. Saini , J. L. Leddon , R. J. Kok , M. Stoff‐Khalili , M. A. Preuss , C. L. Millican , G. Perkins , J. M. Brown , H. Bagaria , D. E. Nikles , D. T. Johnson , V. P. Zharov , D. T. Curiel , Nano Lett. 2006, 6, 587. 16608249 10.1021/nl0500555

[26] J. He , X. Huang , Y. C. Li , Y. Liu , T. Babu , M. A. Aronova , S. J. Wang , Z. Y. Lu , X. Y. Chen , Z. H. Nie , J. Am. Chem. Soc. 2013, 135, 7974. 23642094 10.1021/ja402015s

[27] N. Fomina , J. Sankaranarayanan , A. Almutairi , Adv. Drug Delivery Rev. 2012, 64, 1005. 10.1016/j.addr.2012.02.006 PMC3395781 22386560

