# Supplementary material for: Virus‐Surface‐Mimicking Surface Clustering of AuNPs onto DNA‐Entrapped Polymeric Nanoparticle for Enhanced Cellular Internalization and Nanocluster‐Induced NIR Photothermal Therapy
Source: Adv Sci (Weinh). 2015 Jul 14;2(12):1500108. doi: 10.1002/advs.201500108 (PMC5034825; doi:10.1002/advs.201500108)
Supplement: Supplementary file 1 — Supplementary [file ADVS-2-0c-s001.pdf]

Copyright WILEY-VCH Verlag GmbH & Co. KGaA, 69469 Weinheim, Germany, 2015.

Supporting Information

for *Adv. Sci.*, DOI: 10.1002/advs. 201500108

Virus-Surface-Mimicking Surface Clustering of AuNPs onto DNA-Entrapped Polymeric Nanoparticle for Enhanced Cellular Internalization and Nanocluster-Induced NIR Photothermal Therapy

Hui-Zhen Jia, Wei-Hai Chen, Xuli Wang, Qi Lei, Wei-Na Yin, Yan Wang, Ren-Xi Zhuo, Jun Feng, and Xian-Zheng Zhang*

Copyright WILEY-VCH Verlag GmbH & Co. KGaA, 69469 Weinheim, Germany, 2013.

Supporting Information

Virus-Surface-Mimicking Surface Clustering of AuNPs onto DNA-entrapped Polymeric Nanoparticle for Enhanced Cellular Internalization and Nanocluster-Induced NIR Photothermal Therapy

Hui-Zhen Jia, Wei-Hai Chen, Xuli Wang, Qi Lei, Wei-Na Yin, Yan Wang, Ren-Xi Zhuo, Jun Feng, Xian-Zheng Zhang*

Table of Contents

Table S1. DLS data of PEI1800/DNA and PEI1800/DNA/AuNP……………..……….….....3

Figure S1. Hydrodynamic diameter and Zeta potential of PEI1800/DNA complexes at various N/P ratios………………………………………………………….....4

Figure S2. (A) Transfection efficiency mediated by PEI1800/DNA/AuNP in HeLa, COS7, HepG2 and 3T3 cells. The mass ratio of AuNPs *versus* DNA/vector nanocomplex ranges from 0 to 8; (B) Luciferase assay in HeLa, COS7, HepG2, and 3T3 cells transfected with AuNP-absent (Au-) or AuNP-present (Au+) PEI1800/pGL-3 nanocomplexes. The concentration of pGL-3 was fixed at 1 μg/mL, the N/P ratio was fixed at 20 and the mass ratio of AuNP *versus* DNA/vector nanocomplex was fixed at 7:1……………………………...…....5

Figure S3. Variation of hydrodynamic size and zeta potential of PEI1800/pGL-3 complex upon the addition of AuNPs……………………………………………….…...…6

Figure S4. Cytotoxicity of PEI1800/DNA/AuNP nanocomplexes in HeLa, COS7, HepG2 and 3T3 cells with different w/w ratios of AuNP *versus* DNA/vector nanocomplex. The N/P ratio was fixed at 20 and the concentration of pGL-3 was fixed at 1 μg/mL...7

Materials and Methods……………………………………………………………….....8

- Materials……………………………………………………………………...…8
- Synthesis of gold nanoparticles (AuNPs)……………………………………...…..8

- Transmission Electron Microscopy (TEM)……………………………………………..8
- SEM measurement………………………………………………………………………8
- Amplification and purification of plasmid DNA……………………………………..9
- Cell culture………………………………………………………………………………9
- *In vitro* transfection study by luciferase assay……………………………...…...9
- Particle size and zeta potential measurement……………………………….…....10
- Confocal laser scanning microscopy……………………………………….....…..10
- Flow cytometry analysis…………………………………………………………..…11
- Cytotoxicity assay……………………………………………………………….…..12

References………………………………………………………………………………12

Table S1. DLS data of PEI1800/DNA and PEI1800/DNA/AuNP.

**Sample**	**Size (nm)**	**PDI**	**Zeta potential (mV)**
PEI1800/DNA	110±3.8	0.226	27.0±1.0
PEI1800/DNA/AuNP [a]	225±11	0.190	5.0±0.4

[a] The w/w ratio of AuNPs *versus* DNA/vector nanocomplex was 7:1 and the N/P ratio of PEI1800/DNA was fixed at 20.

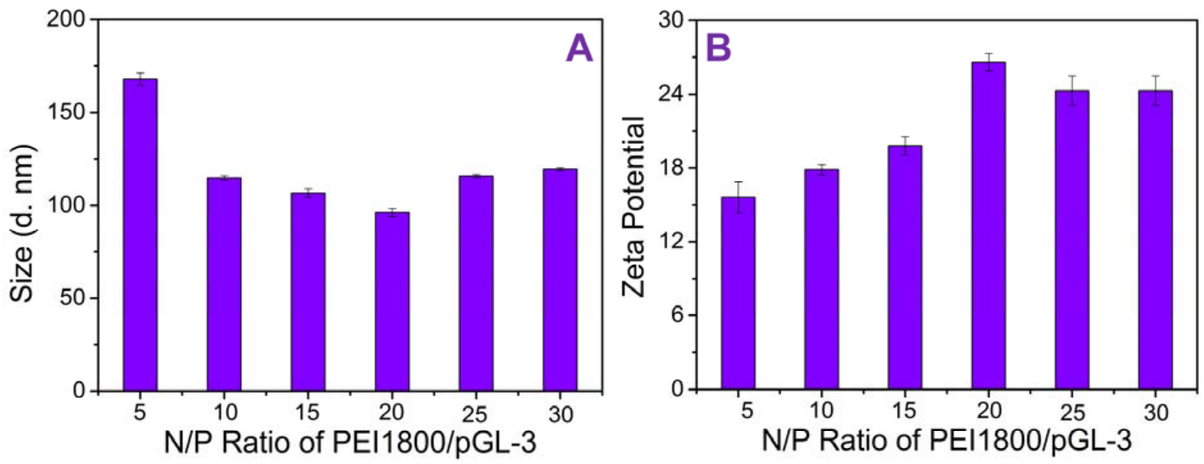

Figure S1. Hydrodynamic diameter and Zeta potential of PEI1800/DNA complexes at various N/P ratios. Data were shown as mean ± SD (n = 3).

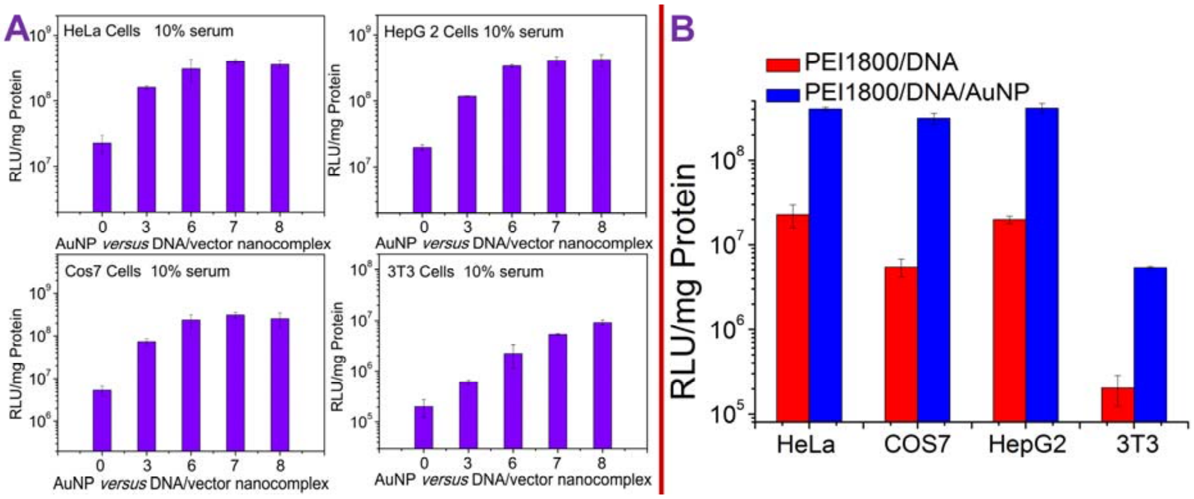

Figure S2. (A) Transfection efficiency mediated by PEI1800/DNA/AuNP in HeLa, COS7, HepG2 and 3T3 cells. The mass ratio of AuNPs *versus* DNA/vector nanocomplex ranges from 0 to 8; (B) Luciferase assay in HeLa, COS7, HepG2, and 3T3 cells transfected with AuNP-absent (Au-) or AuNP-present (Au+) PEI1800/pGL-3 nanocomplexes. The concentration of pGL-3 was fixed at 1 µg/mL, the N/P ratio was fixed at 20 and the mass ratio of AuNP *versus* DNA/vector nanocomplex was fixed at 7:1.

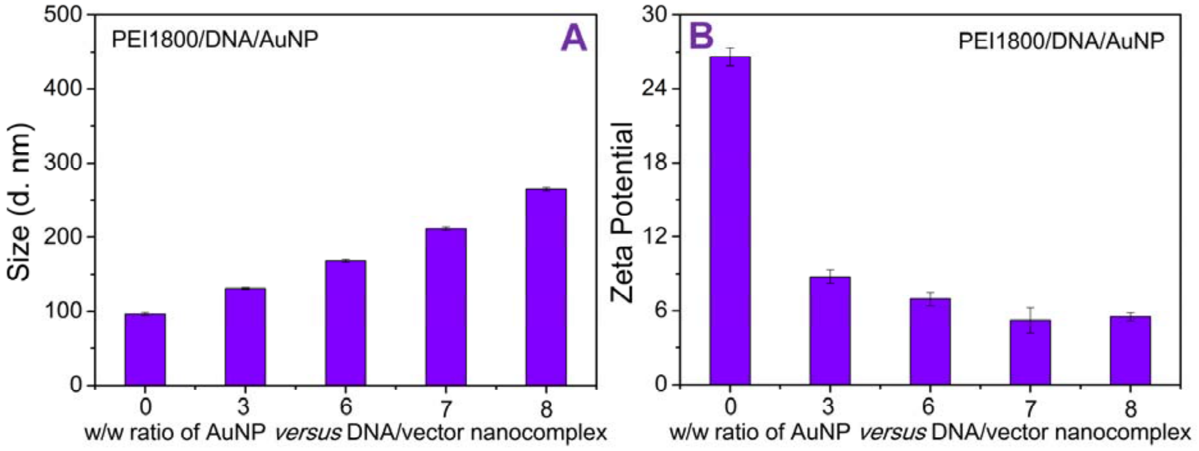

Figure S3. Variation of hydrodynamic size and zeta potential of PEI1800/pGL-3 complex upon the addition of AuNPs. The N/P ratio was fixed at 20. Data were shown as mean ± SD (n = 3).

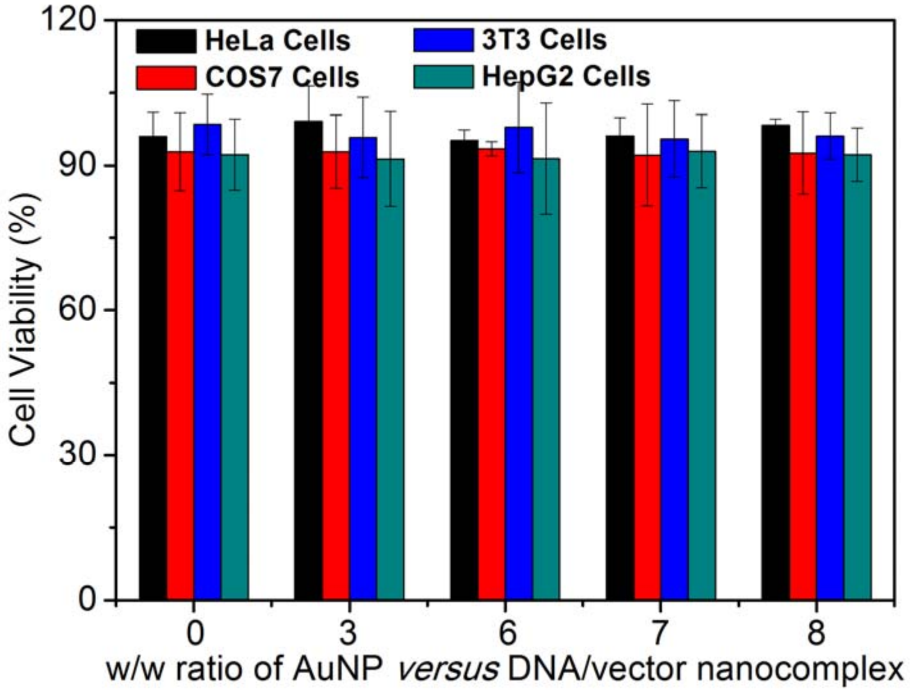

Figure S4. Cytotoxicity of PEI1800/DNA/AuNP nanocomplexes in HeLa, COS7, HepG2 and 3T3 cells with different w/w ratios of AuNP *versus* DNA/vector nanocomplex. The N/P ratio was fixed at 20 and the concentration of pGL-3 was fixed at 1 μg/mL.

Materials and Methods

Materials: polyethyleneimine with the molecular weight of 1800 Dalton (OEI1800) was purchased from Aladdin. Gold(Ⅲ) chloride trihydrate and trisodium citrate dehydrate were obtained from Shanghai Chemical Co. (China) and used directly. Gold nanoparticles (AuNPs) were synthesized according the literatures.[1, 2] Hoechst 33342, Dulbecco's Modified Eagle's Medium (DMEM), 3-[4,5-dimethylthiazol-2-yl]-2,5-diphenyltetrazoliumbromide (MTT), fetal bovine serum (FBS), Dulbecco's phosphate buffered saline (PBS) were purchased from Invitrogen Corp.

Synthesis of gold nanoparticles (AuNPs). The classical Turkevich-Frens method was selected to synthesize the gold nanoparticles (AuNPs). Briefly, 100 mL of 1 mM the $HAuCl_4 \cdot 3H_2O$ was added into the 250 mL round-bottomed flask and refluxed. After boiling, 10 mL of 38.8 mM sodium citrate was rapidly added and the color of the solution was changing from light blue to crimson. After the color changed, the solution was refluxed for another 15 min, and then cooled to room temperature. Subsequently, the solution was filtered using 0.45 μm aqueous phase membrane filter and the gold nanoparticles were prepared.

Transmission Electron Microscopy (TEM). The morphologies of PEI1800/DNA and PEI1800/DNA/Au polyplexes at N/P ratio of 20 were observed by TEM using a JEM-2100 microscope operating at an acceleration voltage of 100 kV. The samples were prepared by dropping the polyplexes solution onto a copper grid with a layer of formvar film before measurement.

SEM measurement. A scanning electron microscope (SEM, JSM-5610LV) was used for studying the exterior morphology of the polyplexes PEI1800/DNA/AuNP. Polyplexes were

prepared as follows: the vector/DNA solution was dropped on a monocrystalline silicon wafer, after the water was volatile completely, the morphologies were observed with SEM.

Amplification and purification of plasmid DNA. pGL-3 plasmid as the luciferase reporter gene and pEGFP-C1 plasmid as the green fluorescent protein expression mediated gene were transformed in Escherichia coli (E. coli) JM109. Each kind of plasmid was amplified in terrific broth medium at 37 ^{o}C overnight. The amplified plasmids were purified by a NucleoBand Xtra Maxi Plus EF kit. Then the plasmids were dissolved in TE buffer solution and stored at -20 ^{o}C. The integrity of plasmids was confirmed by agarose gel electrophoresis, and the purity and concentration of plasmids was determined using a UV spectrophotometer (UV-2401, Shimadzu, Kyoto, Japan) at the wavelengths of 260 and 280 nm.

Cell culture. African Green Monkey SV40-transf'd kidney fibroblast cell line (COS7), human cervix carcinoma (HeLa) cells, human hepatoma (HepG2) cells and mouse fibroblast (3T3) cells were incubated with DMEM containing 1% antibiotics (penicillin-streptomycin, 10000 U mL^{-1}) and 10% FBS at 37 ^{o}C in a humidified atmosphere containing 5% CO_2.

***In vitro* transfection study by luciferase assay.** Transfection of pGL-3 plasmid mediated by PEI1800/DNA/Au was studied in HeLa, COS7, HepG2 and 3T3 cells, and the transfection by PEI1800 served as a positive control. In brief, the cells were seeded in 24-well plates with a density of 6×10^4 cells/well and incubated at 37 ^{o}C for 24 h. Then the polyplexes were prepared with N/P ratio of PEI1800 *versus* pGL-3 was 20, by adding 1 μg pGL-3 to an appropriate volume of PEI1800 solution containing 150 mM NaCl. The polyplex solutions were vortexed for 30 s and incubated at 37 ^{o}C for 30 min. After that, AuNPs were added at w/w ratios of AuNP *versus* DNA/vector nanocomplex ranging from 0 to 8 and vortexed for 30 s. After standing at room temperature for 10 min, the polyplex solutions were diluted to 1 mL

by serum-containing DMEM and added to the cell wells. After a 6-h co-incubation at 37 ^{o}C followed by removing the remaining medium, the cells were washed using PBS. Then 1 mL of fresh DMEM containing 10% FBS was added and the cells were allowed to proliferate for an additional 44 h at 37 ^{o}C. The luciferase assay was performed according to manufacturer's protocol. Relative light units (RLU) were measured by a chemiluminometer (Lumat LB9507, EG&G Berthold, Germany). The total protein was determined according to the BCA protein assay kit (Pierce). Luciferase activity was expressed as RLU/mg protein. Data were shown as mean ± standard deviation (SD) based on three independent measurements.

Particle size and zeta potential measurement. Particle size and zeta potential were determined by dynamic light scattering (DLS) at 25 ^{o}C by Nano-ZS ZEN3600 (Malvern instruments). The polyplexes at N/P ratio of PEI1800 *versus* pGL-3 20 were prepared by adding 1 μg of pGL-3 to an appropriate volume of PEI1800 solution containing 150 mM NaCl. Then the polyplex solution was vortexed for 30 s and incubated for 30 min at 37 ^{o}C. After that, the polyplex solutions were diluted to 1 mL by 150 mM NaCl solution prior to the measurement. While the size and zeta potential of PEI1800/DNA/AuNP with different mass of AuNPs were determined by adding different volume of AuNPs solution (10 nM) into the PEI1800/DNA complexes solution (w/w ratio of AuNP *versus* DNA/vector nanocomplex ranges from 0 to 8) and standing at room temperature for 10 min after vortexing. Data were shown as mean ± standard deviation (SD) based on three independent measurements.

Confocal laser scanning microscopy. Confocal laser scanning microscopy (CLSM) was employed to investigate the cellular uptake of nanoparticles by HeLa, COS7 cells, HepG2 cells and 3T3 cells. The optimal N/P ratio of PEI1800 *versus* pGL-3 at 20 : 1 and the mass ratio of AuNP *versus* DNA/vector nanocomplex at 7:1 detected for the transfection were chosen herein. 1 μg of pGL-3 were fluorescently labeled with 2.5 μL of 1×10^{-5} M YOYO-1

iodide at 37 ^{o}C for 15 min prior to the complexation with polymers. The cells were seeded at a density of 1 × 10^5 cells per well in a single dish and incubated in 1 mL of DMEM containing 10% FBS at 37 ^{o}C for 24 h. The polyplexes were then added in and further incubated for 30 min at 37 ^{o}C. After that, the polyplex solutions were diluted to 1 mL with fresh DMEM containing 10% serum and added immediately to the cells in single dish. Upon 4 h incubation, cells in each dish were washed thrice with 500 μL of PBS. Subsequently, 1 mL of DMEM containing 10 μL Hoechst 33342 was added and then incubated for 15 min at 37 ^{o}C. Prior to observation, the cells were washed thrice with 500 μL of PBS and then photographed under a confocal laser scanning microscope (Nikon C1-si TE2000, Japan, excitation filter 488 nm and emission cut-off filter 515-530 nm for green light). The micrographs of cells were recorded using EZ-C1 Free Viewer 3.70 version software. The whole preparation process should avoid exposure to strong light in order to protect the fluorescent dyes.

Flow cytometry analysis. Flow cytometry was used to quantitatively evaluate the cellular uptake of payloads YOYO-1 labeled pGL-3 in HeLa, COS7, HepG2 and 3T3 cells. In brief, the cells were seeded in the 24-well plates at a density of 5 × 10^4 cells per well and then incubated for 24 h. After that, the medium was replaced with 1 mL of samples solution in fresh DMEM containing 10% serum. Following 4 h incubation, the transfected cells were washed repeatedly with PBS and trypsinized with 0.25% trypsin for 2 min at 37 ^{o}C. Subsequently, the cells were collected and resuspended in 500 μL PBS, then filtrated and examined by flow cytometry (BD FACSAriaTM III, USA). The instrument was calibrated with non-transfected cells (negative control) to identify viable cells, and the transfected cells were determined from a fluorescence scan performed with 1 × 10^4 cells using the FL1-H channel. The concentration of pGL-3 was fixed at 1 μg/mL and the w/w ratio of PEI1800 *versus* pGL-3 was 20. The mass ratio of AuNP *versus* DNA/vector nanocomplex was fixed at 7:1.

Cytotoxicity assay. Cell toxicities of PEI1800/DNA before and after AuNPs loading were examined by MTT assay. The HeLa cells were seeded in 96-well plates at a density of 6.0 × 10^3 cells/well and incubated in 100 μL DMEM containing 10% FBS for 24 h prior to polyplex sample addition. The polymers were complexed with 0.2 μg pGL-3 plasmid at N/P ratio of PEI1800 *versus* pGL-3 20 with different mass ratio of AuNP *versus* DNA/vector nanocomplex in the same way as described in transfection section. For photothermal studies, transfected and non-transfected cells were exposed to a 808 nm CW laser light for 1 min at 2 W/cm^2 after the cells were incubated with the polyplexes for 20 h. Then the medium was replaced with 200 μL of fresh DMEM containing 10% FBS after incubation for 48 h, then 20 μL of MTT (5 mg/mL in PBS buffer) solution was added to each well and further incubated for 4 h at 37 ^{o}C. Subsequently, the medium was removed and 150 μL of DMSO was added. The absorbance intensity at 570 nm was measured using a microplate reader (Bio-Red, Model 550, USA). The relative cell viability was calculated as:

Cell viability (%) = ($OD570_{(smple)}$/$OD570_{(control)}$) × 100%

where $OD570_{(control)}$ and $OD570_{(sample)}$ was obtained in the absence/presence of sample, respectively. Data were shown as mean ± standard deviation (SD) based on three independent measurements.

References

[1] G. Frens, *Nature* **1973**, *241*, 20.

[2] P. A. CooperáStevenson, *Discussions of the Faraday Society* **1951**, *11*, 55.
